# 

**Published:** 1870-08

**Authors:** 


*CONTENTS:*

ORIGINAL COMMUNICATIONS:

ART. I. A Glance at the Rise and Progress of Cell Theories. By I. N. Danforth, M. D. .................. 449

ART. II. Fibrous Tumor of the Bladder Successfully Removed. By A. Reeves Jackson, M. D. .............. 454

ART. III. The Use of the Thermometer in the Diagnosis of Fever. By T. C. Murphy, M. D. ............... 460

ART. IV. Hydrate of Chloral in the Treatment of a Severe Case of Chorea. By J. S. Whitmire, M. D. .... 464

ART. V. Clinical Lecture. By Jno. E. Owens, M. D. Continued ..... 467

ART. VI. Atropia *vs.* Morphia. By Theo. W. Stull, M. D. ............... 471

ART. VII. Death from the Use of "Hair Restorer." Report of Committee, etc. By John W. H. Baker, M. D., Chairman .................. 472

ORIGINAL COMMUNICATIONS (Continued):

ART. VIII. Case of Purulent Pleurisy. By John W. H. Baker, M. D. .. 481

ART. IX. Double Vagina. By J. C. Davis, M. D. ...................... 485

ORIGINAL TRANSLATIONS:

ART. I. Experimental Physiology. Experimental Investigations relative to the Directions of Rotary Movements due to Unilateral Encephalic Lesions. By Dr. J. L. Prevost ............................ 486

EDITOR'S BOOK TABLE ................ 495

EDITORIAL ........................ 503

The American System. Underbidding. Muriatic Acid in Fever. Editors' Convention.

NEWS AND GOSSIP ... ................. 507

